# Longitudinal Molecular Magnetic Resonance Imaging of Endothelial Activation after Severe Traumatic Brain Injury

**DOI:** 10.3390/jcm8081134

**Published:** 2019-07-30

**Authors:** Gloria Vegliante, Daniele Tolomeo, Antoine Drieu, Marina Rubio, Edoardo Micotti, Federico Moro, Denis Vivien, Gianluigi Forloni, Carine Ali, Elisa R. Zanier

**Affiliations:** 1 Department of Neuroscience, Istituto di Ricerche Farmacologiche Mario Negri IRCCS, 20156 Milano, Italy; 2 INSERM, INSERM UMR-S U1237, Physiopathology and Imaging of Neurological Disorders, Normandie University, UNICAEN, Institut Blood and Brain @ Caen-Normandie, GIP Cyceron, 14000 Caen, France; 3 Department of Clinical Research, Caen-Normandie University Hospital, 14000 Caen, France

**Keywords:** traumatic brain injury, neuroimaging, neuroinflammation, endothelial activation

## Abstract

Traumatic brain injury (TBI) is a major cause of death and disability. Despite progress in neurosurgery and critical care, patients still lack a form of neuroprotective treatment that can counteract or attenuate injury progression. Inflammation after TBI is a key modulator of injury progression and neurodegeneration, but its spatiotemporal dissemination is only partially known. In vivo approaches to study post-traumatic inflammation longitudinally are pivotal for monitoring injury progression/recovery and the effectiveness of therapeutic approaches. Here, we provide a minimally invasive, highly sensitive in vivo molecular magnetic resonance imaging (MRI) characterization of endothelial activation associated to neuroinflammatory response after severe TBI in mice, using microparticles of iron oxide targeting P-selectin (MPIOs-α-P-selectin). Strong endothelial activation was detected from 24 h in perilesional regions, including the cortex and hippocampus, and peaked in intensity and diffusion at two days, then partially decreased but persisted up to seven days and was back to baseline 15 days after injury. There was a close correspondence between MPIOs-α-P-selectin signal voids and the P-selectin stained area, confirming maximal endothelial activation at two days. Molecular MRI markers of inflammation may thus represent a useful tool to evaluate in vivo endothelial activation in TBI and monitoring the responses to therapeutic agents targeting vascular activation and permeability.

## 1. Introduction

Traumatic brain injury (TBI) is a leading cause of mortality and morbidity across all ages in all countries, with a bimodal distribution between young adults (13–20 years old) and older adults (>60 years old) [1].

The primary injury resulting from the biomechanical impact directly damages brain tissue by tearing, shearing, and stretching forces. Consequently, a cascade of metabolic, cellular, and molecular events is initiated, leading to secondary damage and contributing largely to the long-term outcome. Although progress in neurosurgery, neuroradiology, and critical care medicine has contributed to the drop of mortality among hospitalized patients [2], no neuroprotective treatments are available [3].

A robust inflammatory response is a key mechanism of secondary injury in TBI [4]. Evidence from animal models and imaging/autopsy studies in humans indicates lasting inflammatory changes and blood-brain barrier (BBB) dysfunction in determining the post-TBI outcome [5]. However, many aspects of the spatial and temporal inflammatory changes in vivo in the human traumatized brain await clarification. Routine imaging techniques (such as computed tomography (CT) and magnetic resonance imaging (MRI)) help clinicians plan surgery and manage complications to secondary injury [6,7] but cannot yet evaluate neuroinflammation. Few probes have been developed to monitor neuroinflammation with positron emission tomography (PET) technique [8]. Moreover, the need for radioactive tracers, high costs and the ability to generate radioligands on-site in the acute phase make the logistics of scanning a major limitation for its clinical use. MRI-based contrast agents (more stable in time and less expensive) for detecting inflammation would be very valuable. Repeated longitudinal studies are feasible since MRI does not need ionizing radiation and can provide images with better spatial resolution than PET.

Micro-sized iron oxide particles (MPIOs) have been proposed to detect the vascular activation in inflammatory conditions, and offer very high sensitivity thanks to their super-paramagnetic properties [9]. Due to their size (~1 µm diameter), MPIOs do not extravasate into the brain parenchyma but can be conjugated to antibodies against specific endothelial adhesion molecules that are over-expressed in brain blood vessels under inflammatory conditions [10,11,12]. We have reported that molecular MRI of P-selectin can reveal discrete, early stages of endothelial dysfunction and can be used to diagnose a transient ischemic attack by disclosing endothelial activation [9,10,11,12,13]. The feasibility of molecular MRI has been proved in other preclinical models of neurological disorders [14], multiple sclerosis [15,16], and brain metastases [17] but has not yet been evaluated for TBI. The aim of this work was to perform a longitudinal study of the brain endothelial activation in a mouse model of severe TBI. The ability to quantify brain-immune interactions at the endothelial level in the TBI brain in vivo would open new avenues for diagnosis, therapeutic monitoring, and understanding the pathogenesis of TBI.

## 2. Experimental Section

### 2.1. Study Approval

The Mario Negri Institute adheres to the principles set out in the following laws, regulations, and policies governing the care and use of laboratory animals: Italian Governing Law (D.lgs 26/2014; Authorization no.19/2008-A issued March 6, 2008 by Ministry of Health); Mario Negri Institutional Regulations and Policies providing internal authorization for persons conducting animal experiments (Quality Management System Certificate – UNI EN ISO 9001:2015 – N°6121); the NIH Guide for the Care and Use of Laboratory Animals (2011 edition) and EU directives and guidelines (EEC Council Directive 2010/63/UE). The Statement of Compliance (Assurance) with the Public Health Service (PHS) Policy on Human Care and Use of Laboratory Animals has been recently reviewed (9/9/2014) and will expire on September 30, 2019 (Animal Welfare Assurance #A5023-01). They were reviewed and approved by the Mario Negri Institute Animal Care and Use Committee which includes ad hoc members for ethical issues, and by the Italian Ministry of Health (Decreto no. D/07/2013-B and 301/2017-PR).

### 2.2. Animals

Male C57BL/6J mice (9–10 weeks old, 20–24 g) from Envigo were used. We followed FELASA guidelines for health monitoring programs. Mice were housed 5 per cage at a constant temperature (21 ± 1 °C) and humidity (60%) on a 12 h light-dark cycle (7 am–7 pm), with ad libitum access to food and water. All animal experiments were designed in accordance with the ARRIVE (Animal Research: Reporting of In Vivo Experiments) guidelines [18], with a commitment to refinement, reduction, and replacement, minimizing the numbers of mice, and using biostatistics to optimize mouse numbers. MRI and histological assessments were done by researchers blinded to the experimental groups.

### 2.3. Experimental Brain Injury

Mice were anesthetized with isoflurane (Merial, Italy) inhalation (induction: 3–4%, maintenance: 1.5–2%) in a N_2_O/O_2_ (70%/30%) mixture, then placed in a stereotaxic frame; local anesthetic (Emla 2.5% lidocaine + 2.5% prilocaine) was used on atraumatic ear bars. An eye lubricant ointment (Lacrigel, Bracco, Italy) was applied to protect corneal membranes during surgery. The skin was disinfected (Clorexyderm 4%, ICF, Italy) before surgical incision. Craniotomy was followed by induction of a single unilateral controlled cortical impact (CCI) brain injury over the left parieto-temporal cortex (AP-2.5 mm, L-2.5 mm). A 3 mm rigid impactor tip electromagnetically driven by a CCI device (Impact One, Leica Biosystems) was applied perpendicularly to the exposed dura mater at an angle of 20°, a velocity of 5 m/s, depth of 2 mm, and a dwell time of 0.1 s [19]. After injury the craniotomy was covered with a cranioplasty and the scalp was sutured. During all surgical procedures, body temperature was maintained at 37°C. Sham mice received identical anesthesia and surgery without brain injury.

### 2.4. MPIOs Preparation

MPIOs (diameter 1.08 µm) with p-toluenesulphonyl (tosyl) reactive surface groups (Dynabeads^®^ MyOne™ Tosylactivated, Invitrogen) were used. Either goat anti-mouse antibodies for P-selectin (AF737; R&D systems, Minneapolis, MN, USA) or normal goat IgG control (AB-108-C; R&D systems, Minneapolis, MN, USA) were covalently conjugated to MPIOs in ammonium sulfate and borate buffer (pH 9.5), by incubation at 37 °C for 48 h. MPIOs were then incubated in buffer containing 0.5% bovine serum albumin (BSA, Sigma Aldrich, Missouri, MO, USA) for 24 h at 37 °C to block the remaining active groups. MPIOs were then rinsed in washing buffer (0.1% BSA) and kept under continuous rolling at 4 °C [14]. Conjugation efficiency with this protocol is reported to be ~10^9^ antibodies per 1 µm MPIO [10]. An equivalent of 2 mg Fe/kg body weight of MPIOs conjugated with either P-selectin or IgG isotype was injected in the tail vein through a cannula, 10 min before MRI (total volume 200 µL/mouse).

### 2.5. MRI Acquisition

**Figure 1:**
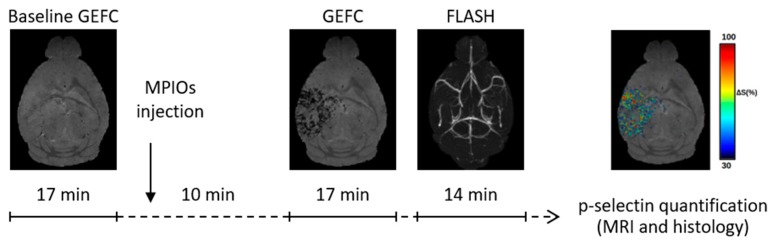
Experimental design. For each mouse a GEFC image was acquired just before (baseline) and 10 min after MPIOs injection. Finally, MRI angiography was examined with a FLASH image. The MRI marked volume was evaluated by measuring the T2* hyposignal occurring after the MPIOs injection and was expressed as the percent change in the signal.

Imaging studies were carried out 1, 2, 3, 5, 7, and 15 days after TBI (3–4 mice/time point). Images were acquired on a 7T Bruker Biospec (Ettlingen, Germany) running ParaVision 6.01, equipped with a quadrature cryogenic surface coil as transmitter and receiver. Mice were anesthetized (induction 3–4%, maintenance 1.5–2% in an air/O_2_-70%/30% mixture) and cannulated in the tail vein for MPIOs infusion. Body temperature was maintained at 37 °C. MPIOs were visualized with a 3D T2*-weighted gradient echo imaging with flow compensation (GEFC) sequence with spatial resolution 80 × 80 × 80 μm^3^, echo time (TE) 7.6 ms, repetition time (TR) of 60 ms and a flip angle (FA) 14°. The acquisition time was 17 min. A GEFC image was acquired before (baseline) and 10 min after MPIOs injection. MRI angiography was also done with a fast low angle shot (FLASH) image with spatial resolution 100 × 100 × 100 μm^3^, TR/TE 19/3.9 ms, FA = 35° and four averages for a scan time of 14 min. MRI acquisition phases are summarized in Figure 1.

### 2.6. MRI Analysis

Contusions are heterogeneous and at early stages include mechanical disrupted tissue, microhemorrhages and edema. Hemorrhages have a strong hypointense signal. Thus to avoid false positives, GEFC images were acquired just before (baseline) and 10 min after MPIOs injection. Changes in the signal were obtained by rigidly aligning each image after MPIOs injection to the relative pre-injection image and the MRI signal void was calculated as the percent signal variation from baseline. The skull-stripping of the brain was done automatically in three steps. (1) A study specific template was created, using the buildtemplateparallel.sh script embedded in the ANTs software library [20], considering all the baseline images. (2) An existing ex-vivo template [21] was normalized over the study-specific template, obtaining the study template brain mask. (3) The mask was finally back-projected to the subject space using the antsIntroduction.sh script [22]. The MRI percent signal change was evaluated as follows:

ΔS(%) = [S(t_1_) − S(t_2_)]/S(t_1_) × 100 (1)

where S(t_1_) and S(t_2_) are respectively the signals before and after MPIOs injection. To suppress the signal-to-noise contribution, a 30% threshold was applied to obtain the marked area. The brain hemispheres were then classified within the brain mask and the marked volume evaluated in the ipsilateral and contralateral regions.

A three-dimensional representation of the marked area was made. The angiographic MRI images were first re-sampled to the relative GEFC images size and skull-stripped by applying the subject brain mask. The 3D-projection of the maximum intensity of the skull-stripped angiography and the image representing the percent signal change was obtained using the image-j software [23]. The 3D-reconstruction of the marked area was computed using the Freesurfer software [24].

### 2.7. Tissue Processing

After MRI imaging, mice were deeply anesthetized with ketamine (20 mg, i.p.) and medetomidine (0.2 mg, i.p.), and transcardially perfused with 30 mL of phosphate-buffered saline 0.1 mol/L (PBS pH 7.4, Lonza, Verviers Belgium), followed by 60 mL of paraformaldehyde 4% (PFA, Merck, Germany) in PBS. The brains were carefully removed from the skull and post-fixed in 4% PFA in PBS for 2 h at 4 °C. The post-fixed tissues were dehydrated with 30% sucrose (Sigma-Aldrich, Missouri, MO, USA) in 0.1 mol/L PBS for 24 h at 4 °C, then frozen in n-pentane for 3 min at −45 °C and stored at −80 °C until used. Serial coronal brain sections (20 µm thick) were cut on a cryostat (+1 mm to −3.5 mm from bregma) at 200 µm intervals for histological analysis.

Immunofluorescence (IF) was performed to detect endothelial (P-selectin), astrocytic (GFAP) and microglia/macrophages (CD68 and IBA1) activation, aquaporin 4 (AQP4) polarization. Tissues were washed and hydrated in PBS solution, then processed as follows:

### 2.8. P-Selectin

Slices were blocked in 0.5% Triton-X100, 5% FBS in PBS. Goat anti-P-selectin (1:300; R&D, clone AF737, Minneapolis, MN, USA) primary antibody was incubated overnight at 4 °C. Donkey anti-goat (1:500, Alexa fluor^488^ or Alexa fluor^647^, Abcam) secondary antibodies were used.

### 2.9. GFAP and AQP4

Slices were blocked in 0.5% Triton-X100, 10% FBS in PBS. Mouse anti-GFAP (1:2000; Chemicon, IL, USA) and rabbit anti-AQP4 (1:1000; Millipore, clone AB3594, California, CA, USA) primary antibodies were incubated overnight at 4 °C. Goat anti-mouse and Goat anti-rabbit (1:500, Alexa fluor^488^ and Alexa fluor^594^ respectively, Abcam) secondary antibodies were used.

### 2.10. IBA1 and CD68

Slices were blocked in 0.3% Triton-X100, 10% NGS in PBS. Rabbit anti-mouse IBA1 (1:200; Wako, 019-19741, Japan) and rat anti-mouse CD68 (1:200; eBioscience, clone FA-11, California, CA, USA) primary antibody were incubated overnight at 4 °C. Goat anti-rabbit and Goat anti-rat (1:500, Alexa fluor^594^ and Alexa fluor^488^ respectively, Abcam) secondary antibodies were used. Negative control studies were run in parallel without the primary antibodies.

### 2.11. Tissue Analysis

Tissues were imaged by Nikon A1 confocal scan unit managed by NIS elements software. Sequential scanning mode was used to avoid bleed-through effects. Eight coronal sections (+0.6 to −2.0 mm from bregma, 400 µm intervals) per mouse were selected to quantify P-selectin stained areas 1, 2 and 7 days after TBI to ensure unbiased, operator-independent sampling. To include all of the P-selectin stained area in the ipsilateral hemisphere throughout the different sections, a 4 × 6 mm region of interest (ROI) was acquired (10× objective, using the large field acquisition command with 10% image overlapping to allow stitching, z-axis 5 µm with a step size of 2 µm). Three sections (+0.4, −1.6, −2.8 mm from bregma) per mouse were acquired to quantify GFAP and AQP4 staining over an area of 300 µm depth from the edge of the contusion (20× objective, 10% overlapping for stitching, z-axis 8 µm with a step size of 2 µm). Representative images were acquired as follows: ROI, 0.5 mm × 0.5 mm, 20× or 40× objective, z-axis 12 µm with a step size of 2 µm. Images were analyzed with Fiji, P-selectin data were plotted as percentages of the stained area, while integrated density was quantified for GFAP and AQP4.

### 2.12. Statistical Methods

Data are expressed as mean + SEM. Statistical analyses were done using GraphPad Prism 7 (GraphPad, San Diego, USA) with a *t*-test for unpaired data or analysis of variance (ANOVA), followed by Tukey’s post-hoc test. *p* < 0.05 was considered statistically significant. No data were excluded from the analysis.

## 3. Results and Discussion

### 3.1. Endothelial Activation Can Be Detected with Molecular MRI Up to One Week Post-TBI

**Figure 2:**
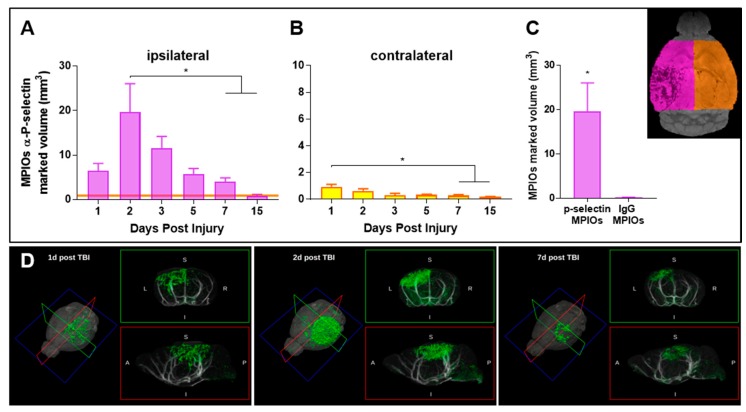
MPIOs-α-P-selectin quantification by MRI. The MPIOs-α-P-selectin marked volume (mm^3^) was longitudinally assessed by MRI up to 15 days post-injury in both the (**A**) ipsilateral (purple, left) and (**B**) contralateral (yellow, right) hemispheres. The orange line in panel A indicates the mean contralateral MPIOs-α-P-selectin marked volume (0.9 mm^3^). Data are mean + SEM, 3-4/group. One-way ANOVA followed by Tukey post-hoc; ipsilateral hemisphere: 2 days vs. 7, 15 days * *p* < 0.05; contralateral hemisphere: 1 day vs. 7, 15 days * *p* < 0.05. (**C**) Hypointense volume (mm^3^) assessed by MRI in ipsilateral hemisphere two days post-TBI in mice injected with MPIOs conjugated with either anti P-selectin or isotypic IgG antibodies. Data are mean + SEM, 3-4/group; unpaired *t*-test, * *p* < 0.05 (**D**) Representative 3D reconstruction with coronal (upper) and sagittal (lower) cross-section of MPIOs-α-P-selectin marked volume (green) 1, 2 and 7 days post-TBI. Vasculature (in white) given by the angiography was co-registered to the T2* MRI images.

We tested whether ultra-sensitive molecular MRI could be an integrative approach in combination with conventional T2-weighted MRI to detect endothelial activation over time in a mouse model of severe TBI. Mice subjected to TBI were intravenously injected with MPIOs coupled to either a control immunoglobulin or to an antibody directed against P-selectin, and scanned at different times after injury for up to two weeks (Figure 2A–D). Specific MPIO-α-P-selectin positive volume doubled from day 1 to day 2 (Figure 2A) with widespread signal voids including the contusional and pericontusional cortex, corpus callosum, hippocampus, choroid plexus, and the medial and lateral thalamus (Figure 2D) on day 2. From day 2 on, MPIOs-α-P-selectin positive volume slowly declined but persisted up to one week. By two weeks, there was no difference in signal between the ipsilateral and contralateral hemispheres. There was also a significant time effect in the contralateral hemisphere, but the signal voids were 10 times lower than in the ipsilateral one (Figure 2B). Signal in sham mice was negligible (Supplementary Figure S1) consistent with previous findings from our group.

No MRI signal void was detectable when mice were injected with untargeted MPIOs (MPIOs-IgG) at the time at which MPIOs-α-P-selectin signal was maximal (two days post-TBI, Figure 2C), confirming the specificity of the signal.

### 3.2. Histological Analysis Highly Reflects Spatial and Temporal Molecular MRI Findings

**Figure 3:**
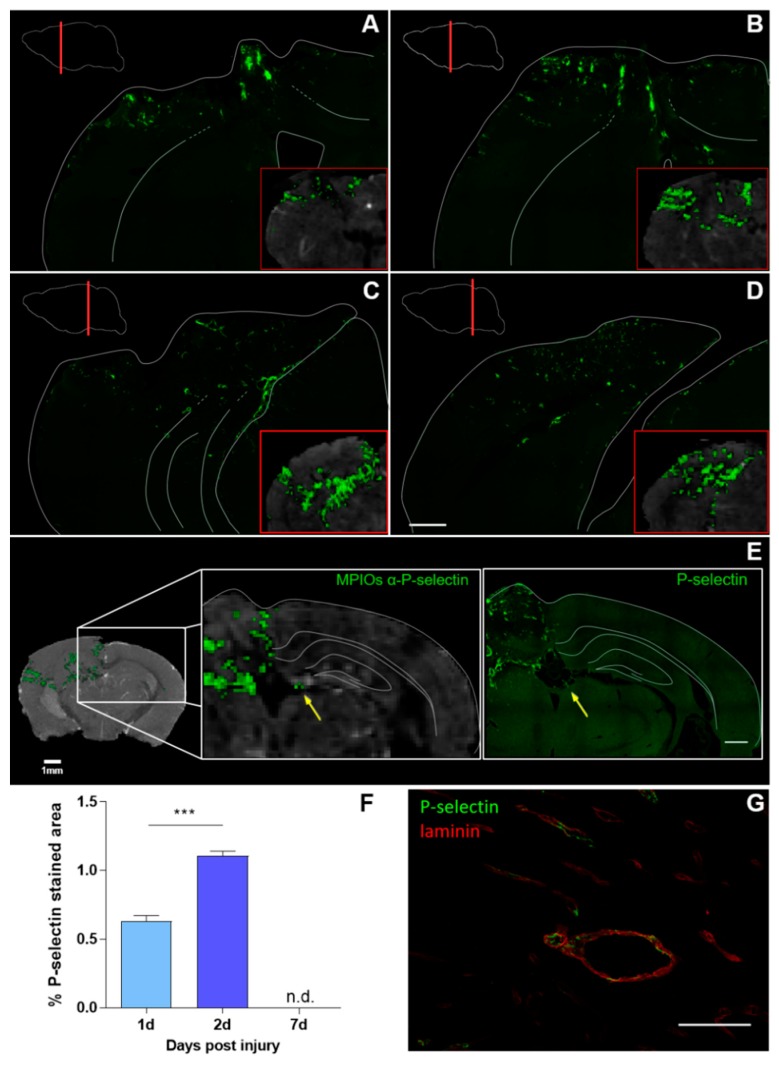
P-selectin signals by MRI and immunofluorescence. (**A**–**D**) Representative IF P-selectin stained coronal sections −0.2, −0.6, −2.4 and 3.2 from bregma and corresponding MRI slices (insert as red boxes) two days post-injury. Scale bar 500 µm. A sketch of the main structures of each brain slice was superimposed on the IF image to facilitate the reading. Data indicate a coherent spatial distribution of P-selectin signal with higher percentage of stained area detected by MRI than with IF. (**E**) Qualitative images showing correspondence between MPIOs-α-P-selectin marked volume assessed by MRI in contralateral hemisphere, and P-selectin IF. Here, the choroid plexus was the only structure ever positive for P-selectin (yellow arrows) by MRI and histological analysis. Scale bar 500 µm (**F**) The percentage of the P-selectin stained area in the ipsilateral hemisphere was assessed 1, 2 and 7 days post-TBI. Data are mean + SEM, 3-4/group; unpaired *t*-test, *** *p* < 0.001. (**G**) P-selectin (green) colocalizes with vessel (laminin in red). Scale bar 100 µm.

Immediately after MRI acquisition, the mice were euthanized and the brain was processed for histology to confirm the spatial and temporal pattern of in vivo imaging findings (Figure 3A–D). There was close correspondence between MPIO α-P-selectin signal voids (red boxes) and P-selectin stained areas throughout the brain. Histological analysis showed a 74.6% increase in P-selectin on day two compared to day one (Figure 3F), mirroring what was seen with molecular MRI. Seven days post-TBI minimal staining was present solely at the contusion edge and only detectable at high (40×) magnification (Figure 3G), thus preventing quantification of P-selectin. The 16 times greater thickness of the MRI over the IF slices may account for the apparent mismatch between MRI and IF signals with the molecular MRI approach providing an in vivo means to detect and quantify endothelial activation even when it is subtle or very confined. MPIOs generate strong magnification of the P-selectin occupied area. The dephasing of the protons extends to a distance ~50 times the size of the particles [14], thus also contributing to the more sensitive MPIO detection than histology.

Consistent with MRI findings, P-selectin staining in the contralateral hemisphere was present only on the choroid plexus (Figure 3E). P-selectin is constitutively expressed on the luminal side of the choroid plexus epithelium and stromal venules [25] and supports continuous trafficking of immune cells and immunosurveillance of the nervous system. Whether the increase indicates recruitment of immune cells in the contralateral hemisphere too calls for further studies.

In the other brain regions, P-selectin colocalized with the vessel marker laminin (Figure 3G). Next to endothelial cells, activated platelet also express P-selectin that mediates their rolling on activated endothelium [26,27]. After TBI, acute platelet dysfunction and hyperaggregation occur [27] possibly contributing to the observed staining or MRI signal voids. However, previous studies in a model of multiple sclerosis, showed similar MRI signal void in platelets depleted compared to non-depleted animals suggesting that MPIOs-α-P-selectin–induced signal void is not influenced by P-selectin expression on platelets [16].

### 3.3. Spatial and Temporal P-Selectin Expression in Relation to Perivascular Disturbances

**Figure 4:**
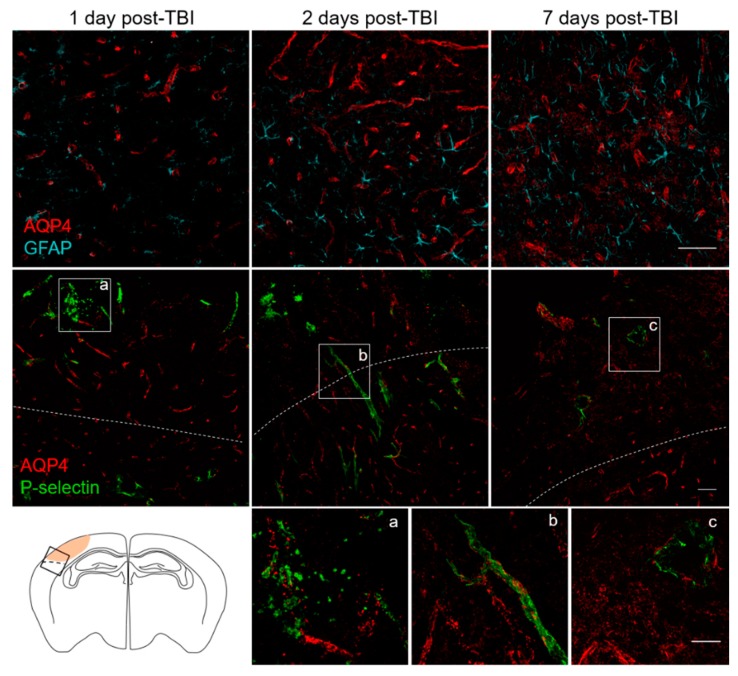
Spatiotemporal distribution of P-selectin in relation to perivascular changes in the acute phase post-TBI. Representative immunofluorescence images for AQP4/GFAP or AQP4/P-selectin staining 1, 2 and 7 days post-TBI at the contusion edge. The white dotted line defines the border between the contusional (upper part of the image) and pericontusional (lower part of the image) tissue, as shown in the sketch. Scale bar 50 µm. Panels (**a**–**c**) are magnification of the AQP4/P-selectin stained areas indicated in the white boxes. Scale bar 25 µm.

In non-pathological conditions, astrocytes are in contact with the brain vasculature constituting the glia limitans. AQP4, clustered predominantly in astrocytic endfeet, contributes to interstitial solute clearance and homeostasis. TBI induces reactive astrocytosis, with changes in expression and localization of AQP4 (Figure 4). AQP4 changes involve a loss of perivascular polarization, defined as the ratio of the focally high perivascular signal area to the overall AQP4 immunoreactivity [28,29], indicating microvascular dysfunction [30,31].

To describe the relationship between P-selectin endothelial expression and alteration/disruption of perivascular cellular function, we performed immunofluorescence for P-selectin, AQP4 and GFAP in mice 1, 2 and 7 days post-TBI (Figure 4). In the ipsilateral cortex, astrocytic activation was associated with a progressive loss of AQP4 polarization over time, up to seven days post-TBI (integrated density: GFAP, increased from 21,242 ± 1017 at 2 days to 30,833 ± 3179 at 7 days, *p* < 0.05; AQP4 decreased from 23,957 ± 961 at 2 days to 19,796 ± 511 at 7 days, *p* < 0.01). There was a difference in the pattern of AQP4 staining at the contusion edge compared to the pericontusional region over time. At early time points (one, two days) in the contusion core where microvessels were disrupted, AQP4 staining was scattered and associated with high positive P-selectin areas. P-selectin staining in this region was still observed at seven days but much reduced, consistent with the P-selectin signal observed by molecular MRI. In the pericontusional area, where perivascular polararization of AQP4 was preserved, P-selectin decorated the vessel lumen on days one and two and was no longer detectable by seven days, consistent with transient, less severe endothelial activation in this region.

**Figure 5:**
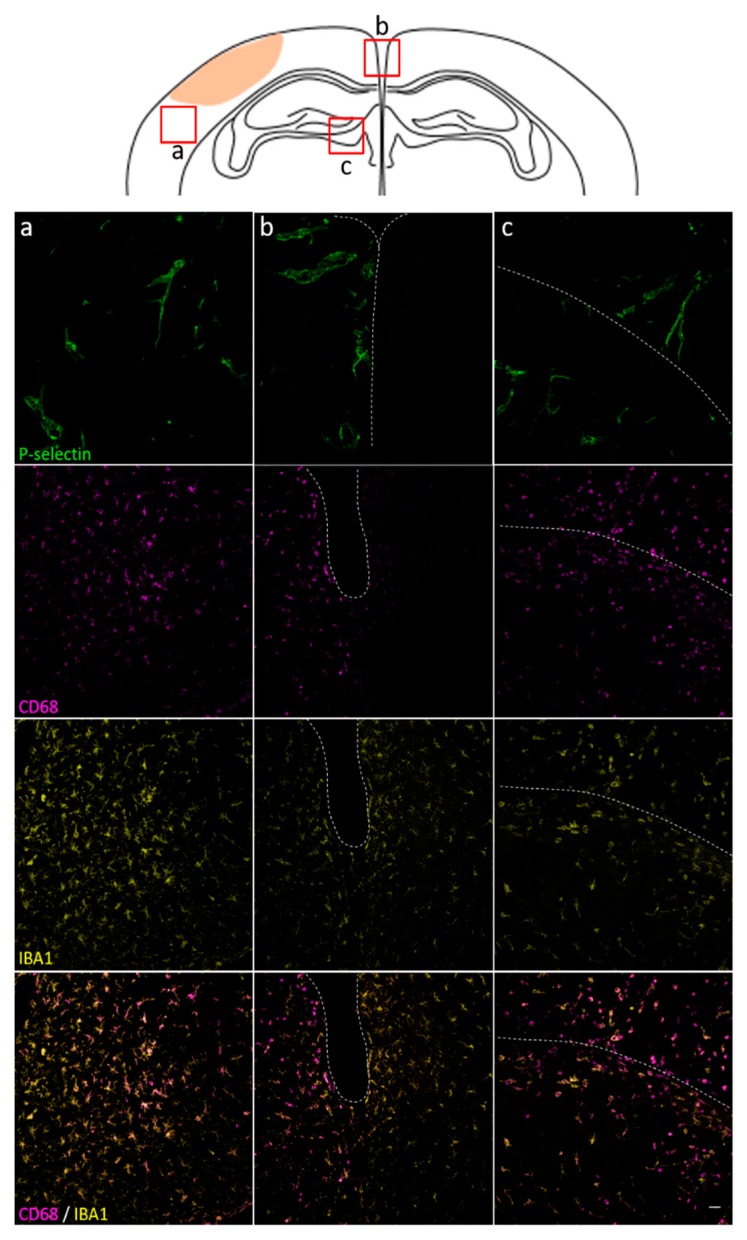
Spatial distribution of microglia/macropahges in relation to P-selectin at 2 days post-TBI. Representative micrographs of P-selectin, CD68, IBA1, and CD68/IBA1 in ipsilateral cortex (**a**,**b**) and hippocampus – thalamus (**c**). The white dotted line defines the border between the hippocampus (upper part of the image) and the thalamus (lower part of the image), as shown in the sketch. TBI induced an increase in P-selectin and CD68 in matched regions and was selectively present in the ipsilateral hemisphere (see also Supplementary Figure S2). Scale bar 50 µm.

To gain insight on the relationship between P-selectin and mircoglia/macrophages activation (M/m) we performed immunofluorescence for IBA1 a pan marker of M/m, and CD68, a marker of lisosomal activity associated to M/m active phagocytosis (Figure 5), two days post-TBI. We observed a spatial concordance between P-selectin (both by conventional IF or molecular MRI) and CD68 positivity with a clear signal at the contusion border, the ipsilateral medial cortex, hippocampus and thalamus, but no contralateral staining (Supplementary Figure S2). Thus, data suggest that targeting P-selectin by in vivo molecular MRI might be useful for investigating in vivo TBI induced endothelial dysfunction not only in the contusion core but also in the spared tissue characterized by ongoing inflammation.

It should be mentioned that unconjugated MPIOs lacking a molecular target, are taken up by circulating immune cells [32]. By contrast, conjugation of MPIOs to antibodies allows an “immediate” antigen/antibody binding, and the conjugated MPIOs stacked on their target will have a strong T2* effect [9]. Thus in our study, where imaging was performed immediately after injection, an aspecific signal due to macrophage MPIOs load is unlikely.

### 3.4. Limitation and Prospects for Molecular In Vivo MRI

This study was designed to obtain a tight temporal description (6 MRI studies within 15 days) of brain endothelial activation after severe TBI by molecular MRI and to confirm the specificity of the results by post-mortem conventional histopathology. For this reason, a longitudinal study within the same mouse was not feasible. It should however be highlighted that having described the peak and time lag of P-selectin activation, future longitudinal studies can be designed taking advantages of the fast clearance (below 24 h) of MPIOs [9]. Among vascular adhesion molecules, P-selectin was chosen based on our previous study showing that MPIOs against P-selectin induced signal voids on MRI even after a transient ischemic attack thus showing the ability to detect subtle endothelial changes, while MPIOs against VCAM1 showed signal voids only after stroke [9]. Additional studies exploring the correlation between other vascular markers like ICAM-1, VCAM-1 or E-Selectin will be of interest.

The main challenges for the years to come are related to the clinical translation of this technique. One of the advantages of iron oxide-based MPIOs is the magnification of the signal (a particle of 1 µm diameter affects a sphere of surrounding tissue of 50 µm in diameter) [14]. However, one of the disadvantages of iron oxide microparticles is that they have negative contrast, similar to pathological conditions such as microbleeding or calcifications. For this reason, caution is needed when interpreting the results in a clinical scenario. The ratio between pre and post injection -as shown in this study- might partially solve this problem. Iron oxide may trigger inflammation, however the iron core (which represents 26% of the total mass) of our MPIOs is enwrapped in a non biodegradable polystyren/polyurethane coating. This structure and composition avoid possible contribution of iron oxide to neuroinflammation.

Additional main steps to be addressed for clinical use were extensively discussed in the review article by Gauberti et al. [14] and include the selection of a suitable pharmacophore; design of a biocompatible MPIO and coating to overcome concerns about potential CNS toxicity of iron oxide compounds [33] and the development of a sensitive MRI sequence to detect MPIOs in humans. In mice, the T2* image has an isotropic resolution of 80 µm, whereas in patients it is 1 mm, an MRI procedure with higher sensitivity is still a pressing need.

Targeting vascular activation and permeability may show promise in TBI, with experimental data showing protective effects of targeted endothelial nanomedicine (i.e., with conjugates of the antioxidant enzyme catalase linked to anti-ICAM-1 antibodies) [34], or repurposing drugs targeting microvascular endothelial cells, i.e., the potent inhibitor of SUR1 TRPM4 (sulfonylureareceptor1 transient receptor potential melastatin 4) channels, Glibenclamide. The latter has been proven effective in the experimental setting in reducing BBB disfunctiom and vasogenic edema [35], and its effect on contusion expansion in TBI patients is under investigation (NCT03954041).

## 4. Conclusions

We have shown that endothelial activation can be detected and monitored in vivo and in situ after TBI by molecular MRI targeting P-selectin, a cell adhesion molecule expressed on the surface of activated endothelial cells. Features of TBI injury include the increase of endothelial cell permeability, heightening the expression of cell-adhesion molecules, and enhancement of immune cell infiltration [36]. These are all key events in the evolution of secondary damage after TBI. Monitoring endothelial activation may thus have diagnostic value. Our data point to potential applications of molecular MRI in severe TBI to assess in vivo the time lag of endothelial activation to monitor the responses to therapeutic agents targeting vascular activation and permeability.

## Supplementary Materials

The following are available online at https://www.mdpi.com/2077-0383/8/8/1134/s1, Figure S1: Sham animal injected with MPIOs-α-P-selectin. Representative coronal slices (A.P.: 0, −1, −1.6, −2 mm from bregma) of a sham showing negligible signal voids 10 minutes after MPIOs-α-P-selectin injection. Figure S2: Spatial distribution of microglia/macropahges in relation to P-selectin 2 days post-TBI in the contralateral hemisphere. Representative micrographs of P-selectin, CD68, IBA1, and CD68/IBA1 in contralateral cortex (a) and hippocampus-thalamus (b). The white dotted line defines the border between the hippocampus (upper part of the image) and the thalamus (lower part of the image), as shown in the sketch. Negligible P-selectin or CD68 staining was detected in the contralateral hemisphere. Scale bar 50 µm.
