# Supplementary material for: Longitudinal Molecular Magnetic Resonance Imaging of Endothelial Activation after Severe Traumatic Brain Injury
Source: J Clin Med. 2019 Jul 30;8(8):1134. doi: 10.3390/jcm8081134 (PMC6722937; doi:10.3390/jcm8081134)
Supplement: Supplementary file 1 [file jcm-08-01134-s001.pdf]

Supplementary Material

Supplementary Figures

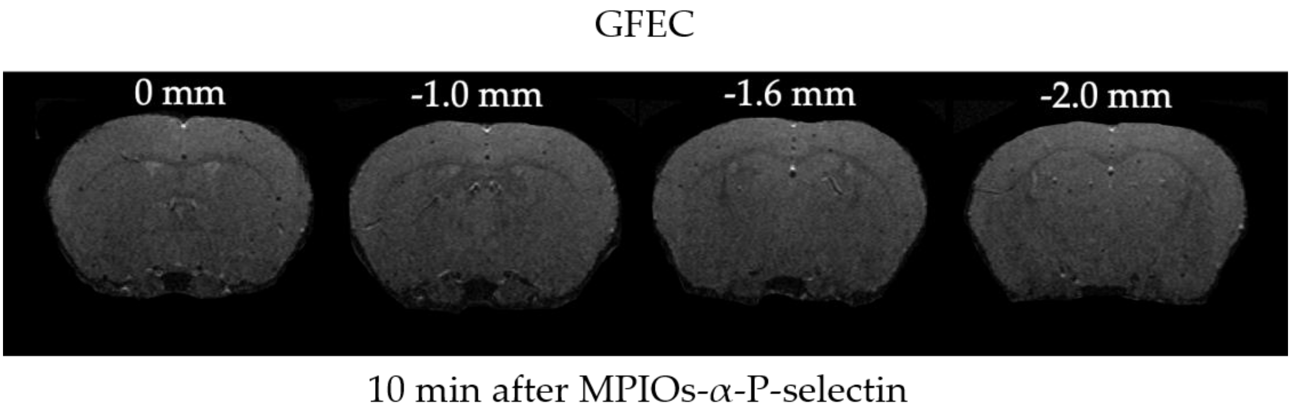

Supplementary Figure 1. Sham animal injected with MPIOs-α-P-selectin. Representative coronal slices (A.P.: 0, -1, -1.6, -2 mm from bregma) of a sham showing negligible signal voids 10 minutes after MPIOs-α-P-selectin injection.

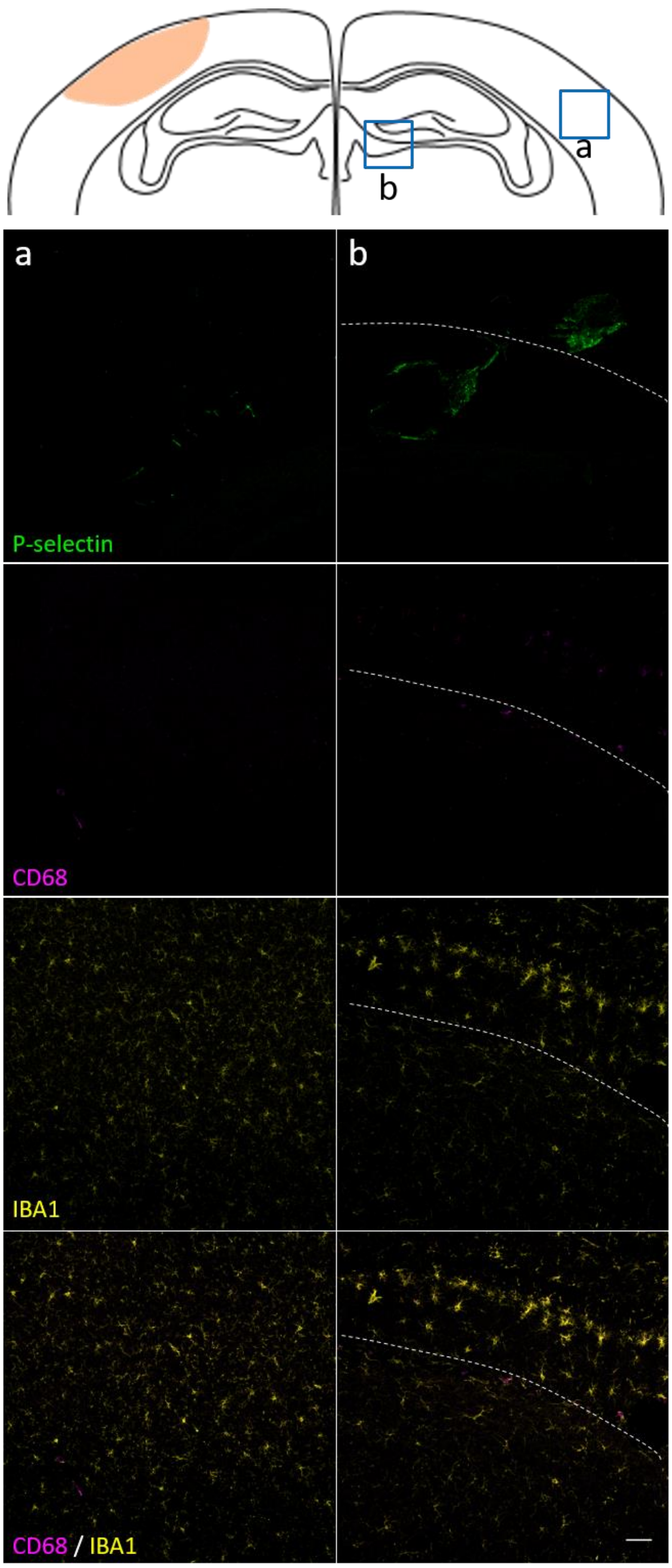

Supplementary Figure 2. Spatial distribution of microglia/macropahges in relation to P-selectin 2 days post-TBI in the contralateral hemisphere. Representative micrographs of P-selectin, CD68, IBA1, and CD68/IBA1 in contralateral cortex (a) and hippocampus-thalamus (b). The white dotted line defines the border between the hippocampus (upper part of the image) and the thalamus (lower part of the image), as shown in the sketch. Negligible P-selectin or CD68 staining was detected in the contralateral hemisphere. Scale bar 50 µm.
